# Supplementary material for: The atypical chemokine receptor ACKR3/CXCR7 is a broad-spectrum scavenger for opioid peptides
Source: Nat Commun. 2020 Jun 19;11:3033. doi: 10.1038/s41467-020-16664-0 (PMC7305236; doi:10.1038/s41467-020-16664-0)
Supplement: Supplementary file 1 — Supplementary Information [file 41467_2020_16664_MOESM1_ESM.pdf]

# The atypical chemokine receptor ACKR3/CXCR7 is a broad-spectrum scavenger for opioid peptides

## Supplementary information

Meyrath, Szpakowska *et al.*

Supplementary Figures 1-8

Supplementary Tables 1-10

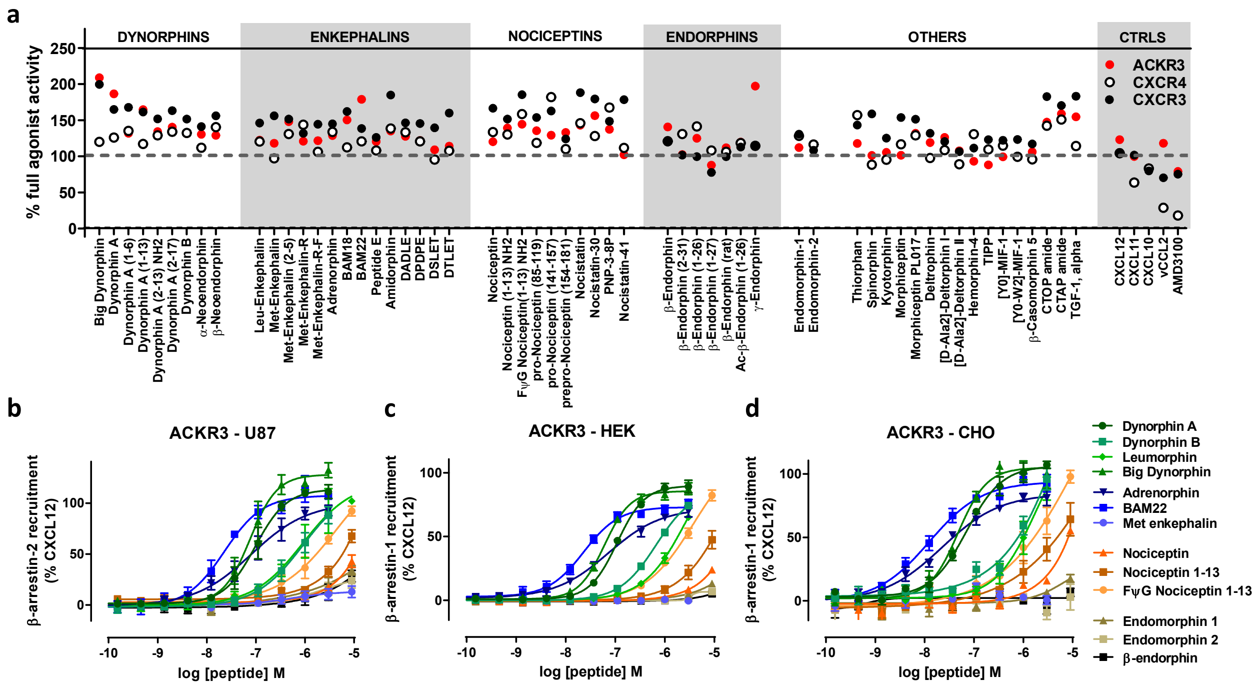


**Supplementary Fig 1. Opioid peptide library screening in antagonist mode and confirmation of ACKR3 activation in different cell lines.** (**a**) Antagonistic activity of 58 compounds including natural opioid peptides from the four opioid families, variants thereof and small molecule opioid receptor modulators (5 µM) towards ACKR3, CXCR4 and CXCR3 evaluated using β-arrestin-2 recruitment assay in U87 cells. For full peptide names and sequences, see supplementary Table 1. Antagonist activity was measured in the presence of CXCL12 (4 nM for ACKR3, 35 nM for CXCR4) or CXCL11 (50 nM for CXCR3) and results are presented as mean of two technical replicates for ACKR3 and CXCR4. A single measurement was performed for CXCR3. (**b-d**) Comparison of potency and efficacy of different opioid peptides in inducing β-arrestin-2 recruitment to ACKR3 in U87 cells **(b)** or β-arrestin-1 recruitment to ACKR3 in HEK cells **(c)** or in CHO cells (**d**). Results are presented as mean ± S.E.M of independent experiments (n = 3-5). The corresponding $EC_{50}$ and Emax values are summarized in supplementary Table 9. Source data are provided as a Source Data file.

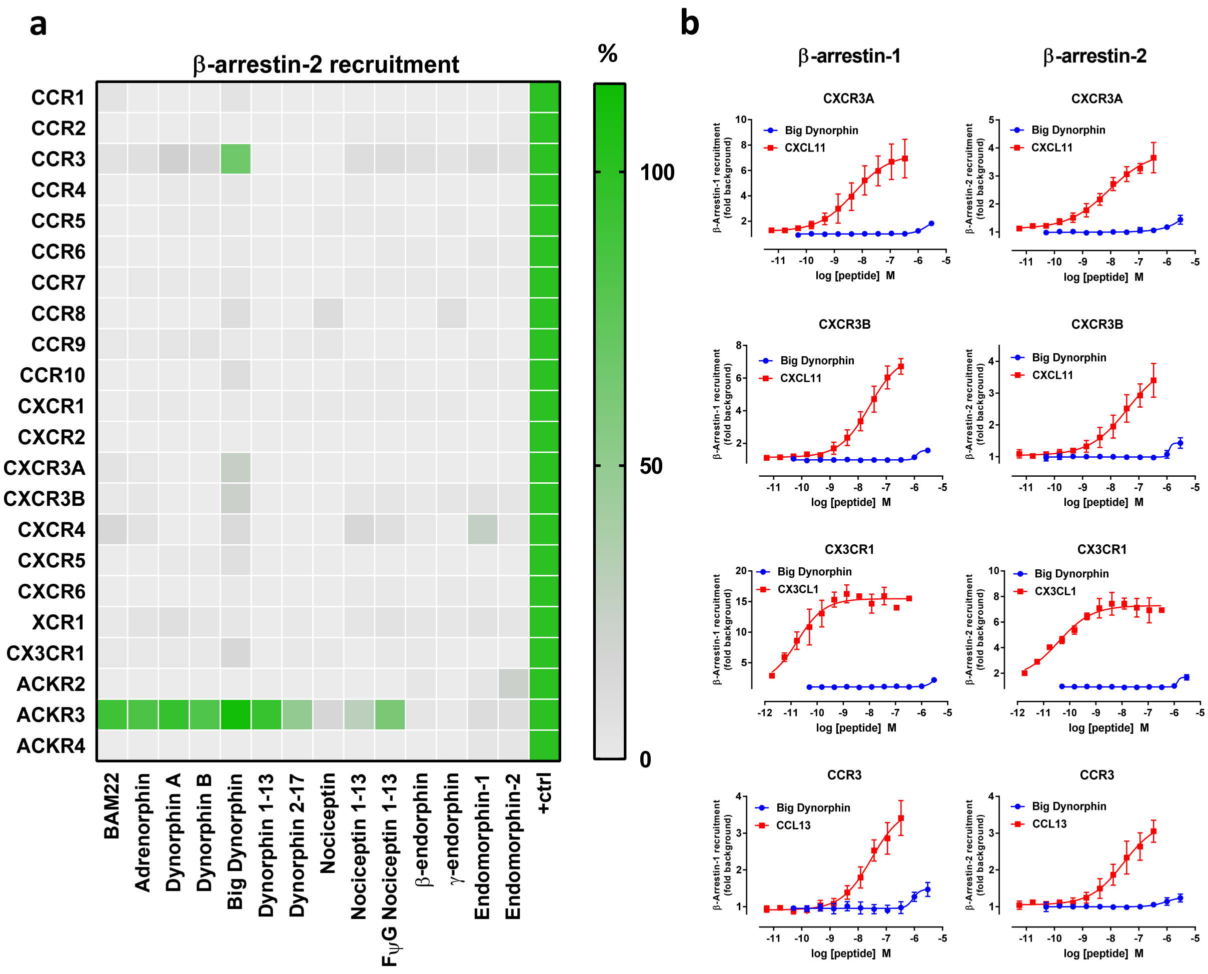


**Supplementary Fig 2. Specific activation of ACKR3 by opioid peptides.** (**a**) Agonist activity of opioid peptides (3 µM) representative of the four opioid families towards 18 classical and 3 atypical chemokine receptors evaluated in β-arrestin-2 recruitment assay. Results are expressed as the percentage of signal recorded with agonist chemokines used as positive controls (supplementary Table 3) at a concentration of 100 nM and presented as mean of independent experiments (n=3) (**b**) Comparison of potency and efficacy of big dynorphin with a full agonist chemokine inducing the recruitment of β-arrestin-1 (left) or β-arrestin-2 (right) to the chemokine receptors CXCR3A, CXCR3B, CX3CR1 or CCR3 in U87 cells. Results are expressed as fold change over untreated and presented as mean ± S.E.M of independent experiments (n =3). The corresponding $EC_{50}$ and Emax values are summarized in supplementary Table 10. Source data are provided as a Source Data file.

a

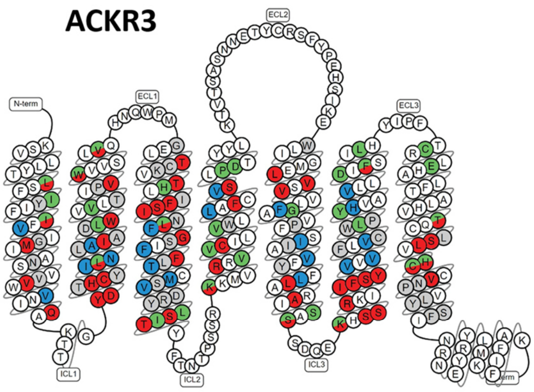


b

| | DOR | KOR | MOR | NOP | ACKR3 | CXCR4 |
|---|---|---|---|---|---|---|
| **TM2** | | | | | | |
| 2x60 | Q105 | Q115 | Q126 | Q107 | W100 | W94 |
| 2x63 | K108 | V118 | N129 | D110 | S103 | D97 |
| **TM3** | | | | | | |
| 3x32 | D128 | D138 | D149 | D130 | F124 | Y116 |
| 3x36 | M132 | M142 | M153 | M134 | L128 | L120 |
| **TM6** | | | | | | |
| 6x23 | S249 | S262 | S270 | S251 | - | - |
| 6x24 | K250 | R263 | K271 | R252 | - | - |
| 6x25 | E251 | E264 | E272 | E253 | - | - |
| 6x26 | K252 | K265 | K273 | D255 | - | - |
| 6x27 | D253 | D266 | D274 | R256 | - | - |
| 6x28 | R254 | R267 | R275 | R256 | - | - |
| 6x29 | S255 | N268 | N276 | N257 | - | - |
| 6x52 | H278 | H291 | H299 | Q280 | H269 | Y256 |
| 6x58 | W284 | E297 | K305 | Q286 | D275 | D262 |
| **TM7** | | | | | | |
| 7x23 | - | - | - | - | T286 | G273 |
| 7x24 | - | - | - | - | C287 | C274 |
| 7x25 | - | - | - | - | R288 | E275 |
| 7x26 | - | - | - | - | L289 | F276 |
| 7x27 | - | - | - | - | E290 | E277 |
| 7x34 | L300 | Y312 | W320 | L301 | L297 | I284 |
| 7x38 | I304 | I316 | I324 | T305 | Q301 | E288 |
| 7x45 | N310 | N322 | N330 | N311 | H307 | H294 |

c

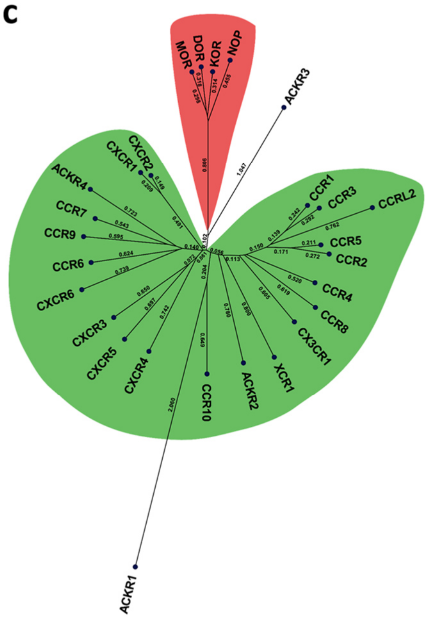


**Supplementary Fig 3. Phylogenetic analysis and comparison of residues conserved in ACKR3, opioid and chemokine receptors.** (**a**) Snake diagram depicting ACKR3 topology and identifying the positions occupied by residues conserved only in ACKR3 and CXCR4 (green), conserved in opioid receptors but not in ACKR3 (red), conserved separately in chemokine receptors and opioid receptors (green /red), conserved in at least three of the four opioid receptors and ACKR3 (blue) or in opioid receptors as well as ACKR3 and CXCR4 (grey) (GPCRdb). (**b**) Comparison of particular positions such as positions delineating the orthosteric binding pocket of opioid receptors (2x60, 3x32, 3x36, 6x52, 7x34, 7x38 and 7x45) or CXCR4 (2x63, 6x58 and 7x38), the bottom of TM6 (6x23 to 6x29), the top of TM7 (7x23 to 7x27) or key conserved position in chemokine receptors (7x38). (**c**) Phylogenetic tree of the human chemokine and opioid receptors showing that ACKR3 stands on a separate branch in between the chemokine and the opioid receptors (GPCRdb).

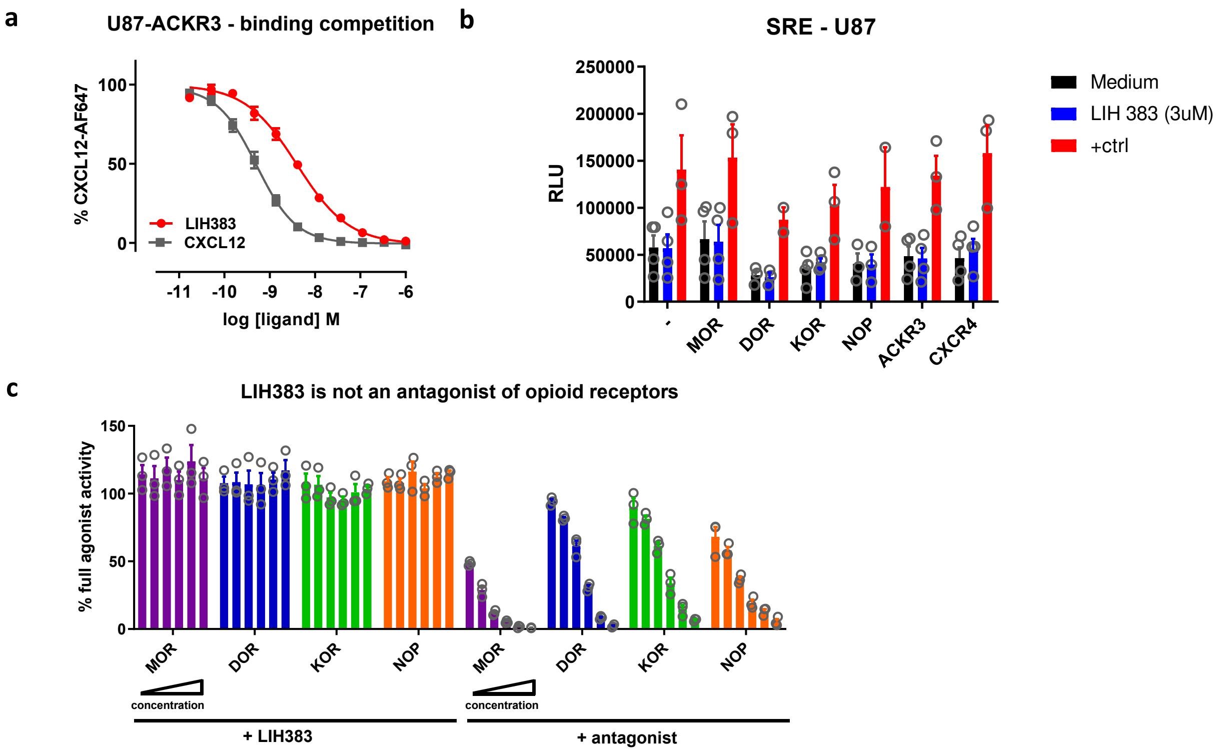


**Supplementary Fig 4. Absence of activity and signaling by LIH383 towards classical opioid receptors and ACKR3-binding potency of LIH383.** (**a**) Binding competition of increasing concentrations of ACKR3-activating CXCL12 or LIH383 with Alexa Fluor 647-labelled CXCL12 (5 nM) on U87-ACKR3 cells determined by flow cytometry. (**b**) Activation of SRE (ERK1/2) signaling cascade in U87 cells (-) or U87 expressing ACKR3, CXCR4 or classical opioid receptors (MOR, DOR, KOR or NOP) in response to 3 µM LIH383 or positive controls (+ctrl, 30 nM PMA, 10 % FBS). Medium only was used as reference. (**c**) Dose dependent antagonist activity of LIH383 (12.3-3000 nM) towards classical opioid receptors MOR, DOR, KOR and NOP determined by β-arrestin-1 recruitment. Naloxone (4.1-1000 nM) for MOR, DOR and KOR or buprenorphine (111-27000 nM) for NOP were used as positive control antagonists. Antagonist activity was measured following addition of BAM22 (50 nM), met-enkephalin (70 nM), dynorphin A (50 nM), nociceptin (70 nM) on MOR, DOR, KOR and NOP, respectively. Results are presented as mean ± S.E.M of independent experiments (n = 3-5). Source data are provided as a Source Data file.

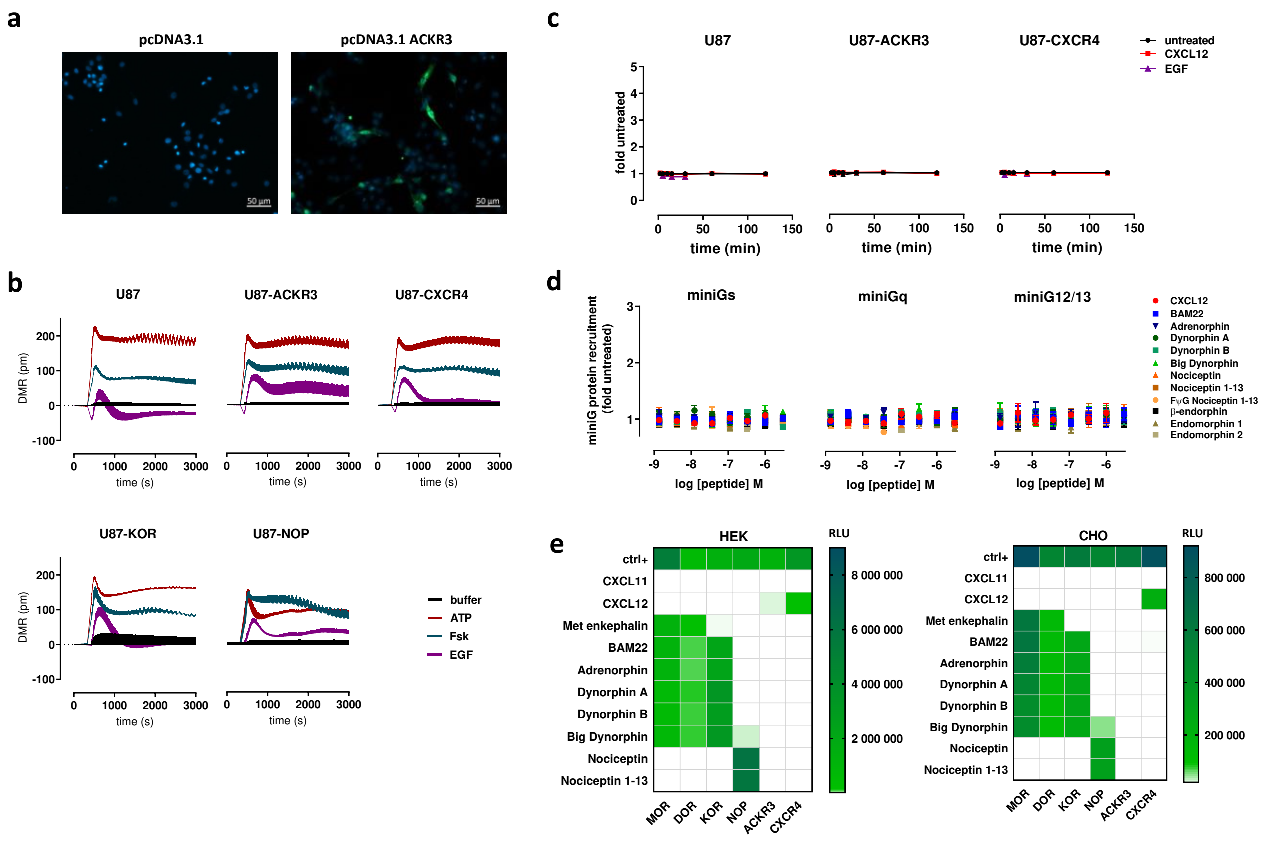


**Supplementary Fig 5. Absence of biased/alternative ACKR3 signaling in response to opioid peptides in different cell lines.** (**a**) Verification of effective ACKR3 transfection by fluorescence microscopy for DMR experiments. Adherent U87 cells transfected with pcDNA3.1-ACKR3 or an empty pcDNA3.1 vector were fixed and stained with an ACKR3-specific primary mouse antibody (clone 11G8) and a secondary Cy2-conjugated anti-mouse antibody (green). Nuclei were stained with DAPI (blue). Pictures are representative of three independent transfections used for DMR experiments. Scale bar: 50µm. (**b**) DMR profiles of U87 cells expressing (or not) ACKR3, CXCR4 or classical opioid receptors (KOR and NOP) stimulated with 100 µM of adenosine triphosphate (ATP), forskolin (Fsk) or epidermal growth factor (EGF) determined over 3000 seconds. Representative profiles of three to five independent experiments are shown. (**c**) Kinetic analysis of total ERK in U87 cells stably expressing ACKR3 or CXCR4 stimulated by CXCL12 or EGF. Results represent the mean of independent experiments (n=2). **(d)** mini Gs, mini Gq and miniG12/13 recruitment to ACKR3 in response to CXCL12 and opioid peptides monitored in U87 cells. Results represent the mean ± S.E.M of independent experiments (n = 3-5). (**e**) Activation of SRE (ERK1/2) signaling cascade in HEK (left panel) or CHO (right panel) cells expressing or not ACKR3, CXCR4 or classical opioid receptors MOR DOR KOR or NOP in response to chemokines CXCL12 and CXCL11, opioid peptides or positive controls (30 nM PMA, 10 % FBS). Results represent the mean of independent experiments (n = 3). Source data are provided as a Source Data file.

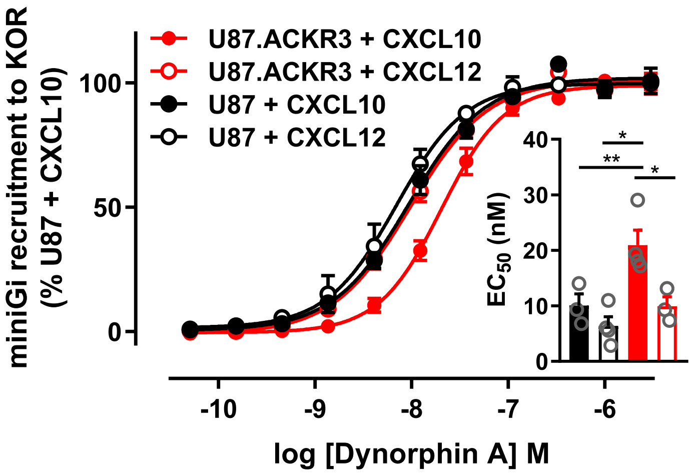


**Supplementary Fig 6. Regulation by ACKR3 of opioid peptide availability for classical receptors.** Dynorphin A-induced concentration-dependent recruitment of mini Gi to KOR in the presence of tenfold excess of U87 or U87-ACKR3 cells (1.5 x $10^5$) pre-treated for 15 minutes with CXCL12 (200nM) to block ACKR3, or the irrelevant chemokine CXCL10 (200nM). The determined $EC_{50}$ values are represented as bars in the inset. Results are presented as mean ± S.E.M of independent experiments (n=3 for U87 + CXCL10 and U87 ACKR3 + CXCL12 and n=4 for both other conditions). * $p< 0.05$, ** $p< 0.01$ by Two-way ANOVA: interaction between cell line and chemokine treatment with Tukey's post hoc test. Source data and statistical analysis parameters are provided as a Source Data file.

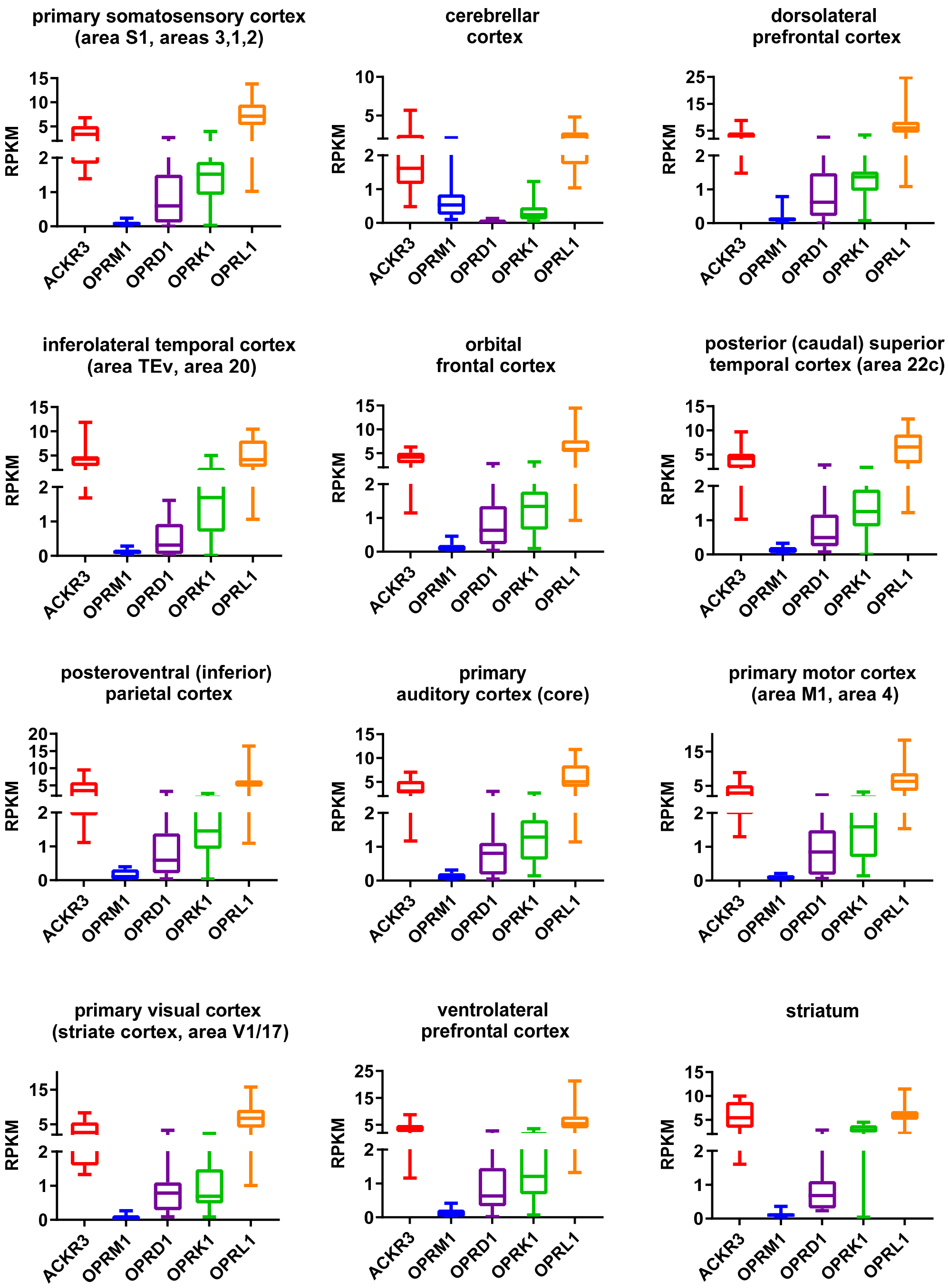


**Supplementary Fig 7. Relative gene expression of ACKR3 and classical opioid receptors in different brain regions.** RNA-Seq RPKM (reads per kilobase per million) values from the open-source brainspan.org database containing data of different brain regions from 14–22 donors (n=14 for striatum, n=15 for dorsolateral prefrontal cortex, n=16 for primary somatosensory cortex and for primary motor cortex, n=17 for orbital frontal cortex and for primary auditory cortex, n=18 for primary visual cortex, n=19 for ventrolateral prefrontal cortex and for posteroventral parietal cortex, n=21 for cerebrellar cortex and for inferolateral temporal cortex, n=22 for posterior superior temporal cortex). Box plots encompass 25$^{th}$ to 75$^{th}$ percentile with median as central mark and whiskers extending to most extreme data points.

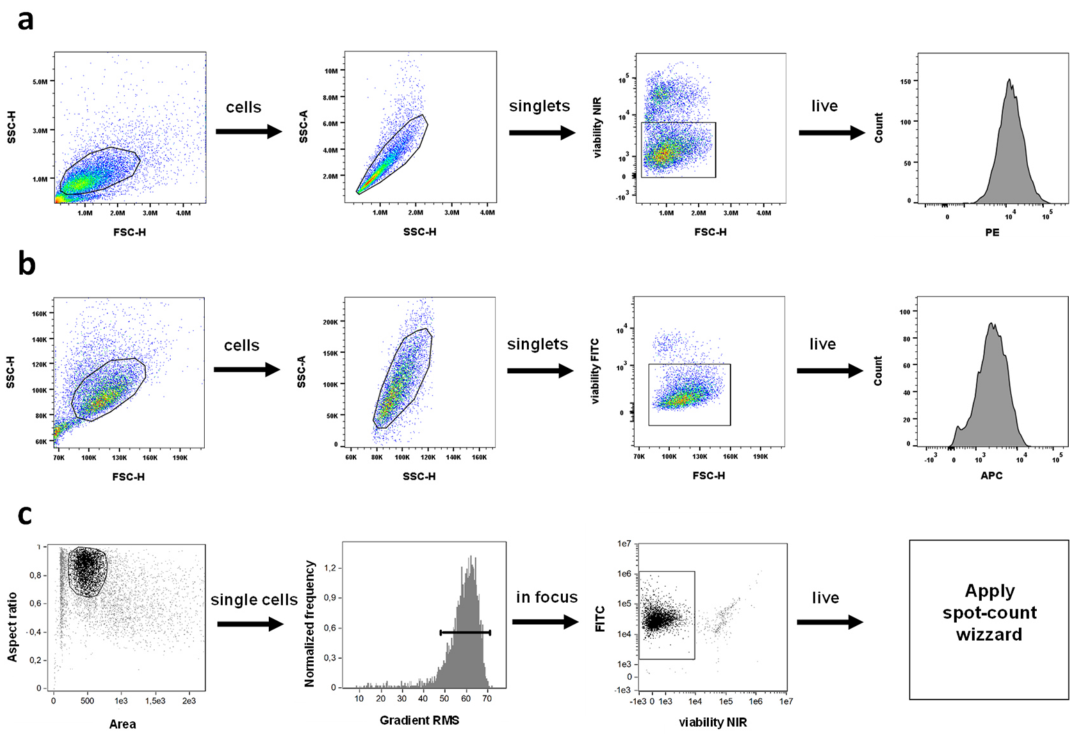


**Supplementary Fig 8. Gating strategy used in experiments to determine receptor surface expression, LIH383-Cy5 and CXCL12-AF647 binding and opioid peptide internalization. (a)** Gating strategy for two-color flow cytometry analysis of receptor surface expression reported in figure 6g. After ligand treatment followed by an acid wash, cells were incubated with receptor-specific mAbs (clones 11G8 for ACKR3 and 387301 for KOR) and a secondary PE-conjugated F(ab')$_2$ fragment anti-mouse IgG. Dead cells were excluded using a Zombie NIR viability dye (near infrared). **(b)** Gating strategy for two-color flow cytometry analysis to determine CXCL12-AF647 or LIH-Cy5 binding presented in figure 1g, figure 4f and supplementary figure 4a. Cells were treated with either LIH383-Cy5 or a mixture of CXCL12-AF647 and unlabeled ligand. Dead cells were excluded with Zombie Green viability dye (FITC) and signal was recorded in APC channel. **(c)** Gating strategy for two-color imaging flow cytometry to evaluate opioid peptide uptake and presence of individual intracellular fluorescent spots represented in figure 6a, figure 6b and figure 7e. Gates were set to include only single, in-focus cells. Dead cells were excluded with the Zombie NIR viability dye for FAM-labeled peptides or with Zombie Green viability dye for Cy5-labeled peptides. Geometrical MFI from FITC channel for FAM-labeled peptides, or from APC channel for Cy-5 labeled peptides was determined. The number of spots per cell was determined using a mask-based software wizard.

**Supplementary Table 1. Sequences, full names and family of endogenous opioid peptides and variants thereof used in experiments reported in Figure 1A and supplementary Figure 1A.**

| | Name/Abbreviation used in Fig 1A | Full name and species (as indicated by phoenixpeptide.com) | sequence (three letter code) |
|---|---|---|---|
| Dynorphins | Big Dynorphin | Dynorphin, big (Human, Rat, Mouse, Porcine) | Tyr-Gly-Gly-Phe-Leu-Arg-Arg-Ile-Arg-Pro-Lys-Leu-Lys-Trp-Asp-Asn-Gln-Lys-Arg-Tyr-Gly-Gly-Phe-Leu-Arg-Arg-Gln-Phe-Lys-Val-Val-Thr |
| | Dynorphin A | Dynorphin A (Human, Rat, Mouse, Porcine) | Tyr-Gly-Gly-Phe-Leu-Arg-Arg-Ile-Arg-Pro-Lys-Leu-Lys-Trp-Asp-Asn-Gln |
| | Dynorphin A (1-6) | Dynorphin A (1-6) / Endorphin (1-6), alpha-Neo / Leu-Enkephalin-Arg (Human, Rat, Mouse, Porcine) | Tyr-Gly-Gly-Phe-Leu-Arg |
| | Dynorphin A (1-13) | Dynorphin A (1-13) (Human, Rat, Mouse, Porcine) | Tyr-Gly-Gly-Phe-Leu-Arg-Arg-Ile-Arg-Pro-Lys-Leu-Lys |
| | Dynorphin A (2-13) NH2 | Dynorphin A (2-13) amide (Human, Rat, Mouse, Porcine) | Gly-Gly-Phe-Leu-Arg-Arg-Ile-Arg-Pro-Lys-Leu-Lys-NH2 |
| | Dynorphin A (2-17) | Dynorphin A (2-17) (Human, Rat, Mouse, Porcine) | Gly-Gly-Phe-Leu-Arg-Arg-Ile-Arg-Pro-Lys-Leu-Lys-Trp-Asp-Asn-Gln |
| | Dynorphin B | Dynorphin B / Rimorphin (Human, Rat, Mouse, Porcine) | Tyr-Gly-Gly-Phe-Leu-Arg-Arg-Gln-Phe-Lys-Val-Val-Thr |
| | α-Neoendorphin | Endorphin, alpha-Neo (Porcine) | Tyr-Gly-Gly-Phe-Leu-Arg-Lys-Tyr-Pro-Lys |
| | β-Neoendorphin | Endorphin, beta-neo | Tyr-Gly-Gly-Phe-Leu-Arg-Lys-Tyr-Pro |
| Enkephalins | Leu-Enkephalin | Leu-Enkephalin | Tyr-Gly-Gly-Phe-Leu |
| | Met-Enkephalin | Met-Enkephalin | Tyr-Gly-Gly-Phe-Met |
| | Met-Enkephalin (2-5) | [Des-Tyr1]-Met-Enkephalin | Gly-Gly-Phe-Met |
| | Met-Enkephalin-R | Met-Enkephalin-Arg | Tyr-Gly-Gly-Phe-Met-Arg |
| | Met-Enkephalin-R-F | Met-Enkephalin-Arg-Phe | Tyr-Gly-Gly-Phe-Met-Arg-Phe |
| | Adrenorphin | Metorphinamide / Adrenorphin | Tyr-Gly-Gly-Phe-Met-Arg-Arg-Val-NH2 |
| | BAM18 | BAM-18P (Bovine) | Tyr-Gly-Gly-Phe-Met-Arg-Arg-Val-Gly-Arg-Pro-Glu-Trp-Trp-Met-Asp-Tyr-Gln |
| | BAM22 | BAM-22P (Bovine) | Tyr-Gly-Gly-Phe-Met-Arg-Arg-Val-Gly-Arg-Pro-Glu-Trp-Trp-Met-Asp-Tyr-Gln-Lys-Arg-Tyr-Gly |
| | Peptide E | Peptide E / 3200-Dalton Adrenal Enkephalin-Containing Peptide (Bovine) | Tyr-Gly-Gly-Phe-Met-Arg-Arg-Val-Gly-Arg-Pro-Glu-Trp-Trp-Met-Asp-Tyr-Gln-Lys-Arg-Tyr-Gly-Gly-Phe-Leu |
| | Amidorphin | pro-Enkephalin A (104-129) amide / Amidorphin (Bovine) | Tyr-Gly-Gly-Phe-Met-Lys-Lys-Met-Asp-Glu-Leu-Tyr-Pro-Leu-Glu-Val-Glu-Glu-Glu-Ala-Asn-Gly-Gly-Glu-Val-Leu-NH2 |
| | DADLE | [D-Ala2, D-Leu5]-Enkephalin | Tyr-D-Ala-Gly-Phe-D-Leu |
| | DPDPE | [D-Pen2, D-Pen5]-Enkephalin | Tyr-D-Pen-Gly-Phe-D-Pen Disulfide bridge: Pen2-Pen5 |
| | DSLET | [D-Ser2]-Leu-Enkephalin-Thr / Delta-Receptor Peptide / DSLET | Tyr-D-Ser-Gly-Phe-Leu-Thr |
| | DTLET | [D-Thr2]-Leu-Enkephalin-Thr / DTLET | Tyr-D-Thr-Gly-Phe-Leu-Thr |
| Nociceptins | Nociceptin | Orphanin FQ / Nociceptin (Human, Rat, Mouse, Ox) | Phe-Gly-Gly-Phe-Thr-Gly-Ala-Arg-Lys-Ser-Ala-Arg-Lys-Leu-Ala-Asn-Gln |
| | Nociceptin (1-13) NH2 | Orphanin FQ (1-13) amide / Nociceptin (1-13) amide (Human, Rat, Mouse, Ox) | Phe-Gly-Gly-Phe-Thr-Gly-Ala-Arg-Lys-Ser-Ala-Arg-Lys-NH2 |
| | [F-ψ-G]Nociceptin(1-13) NH2 | [Phe-psi-Gly]-Orphanin FQ (1-13) amide / [Phe-psi-Gly]-Nociceptin (1-13) amide (Human, Rat, Mouse, Ox) | [Phe1ψ(CH2-NH)-Gly2]-Gly-Phe-Thr-Gly-Ala-Arg-Lys-Ser-Ala-Arg-Lys-NH2 |
| | pro-Nociceptin (85-119) | pro-Orphanin FQ (85-119) / pro-Nociceptin (85-119) / Nocistatin-35 (Rat) | Met-Pro-Arg-Val-Arg-Ser-Val-Val-Gln-Ala-Arg-Asp-Ala-Glu-Pro-Glu-Ala-Asp-Ala-Glu-Pro-Val-Ala-Asp-Glu-Ala-Asp-Glu-Val-Glu-Gln-Lys-Gln-Leu-Gln |
| | pro-Nociceptin (141-157) | pro-Orphanin FQ (141-157) / pro-Nociceptin (141-157) | Phe-Ser-Glu-Phe-Met-Arg-Gln-Tyr-Leu-Val-Leu-Ser-Met-Gln-Ser-Ser-Gln |
| | prepro-Nociceptin (154-181) | prepro-Orphanin FQ (154-181), free acid (Rat) / prepro-Orphanin FQ (160-187) free acid (Mouse) | Phe-Ser-Glu-Phe-Met-Arg-Gln-Tyr-Leu-Val-Leu-Ser-Met-Gln-Ser-Ser-Gln-Arg-Arg-Arg-Thr-Leu-His-Gln-Asn-Gly-Asn-Val |
| | Nocistatin | prepro-Orphanin FQ (111-127) / Nocistatin / PNP-3 (Bovine) | Thr-Glu-Pro-Gly-Leu-Glu-Glu-Val-Gly-Glu-Ile-Glu-Gln-Lys-Gln-Leu-Gln |
| | Nocistatin-30 | prepro-Orphanin FQ (98-127) / Nocistatin-30 (Human) | Met-Pro-Arg-Val-Arg-Ser-Leu-Phe-Gln-Glu-Gln-Glu-Glu-Pro-Glu-Pro-Gly-Met-Glu-Glu-Ala-Gly-Glu-Met-Glu-Gln-Lys-Gln-Leu-Gln- |
| | PNP-3-8P | PNP-3-8P (Bovine) | Glu-Ile-Glu-Gln-Lys-Gln-Leu-Gln |
| | Nocistatin-41 | PNP-2/3 / Nocistatin-41 (Mouse) | Met-Pro-Arg-Val-Arg-Ser-Leu-Val-Gln-Val-Arg-Asp-Ala-Glu-Pro-Gly-Ala-Asp-Ala-Glu-Pro-Gly-Ala-Asp-Ala-Glu-Pro-Gly-Ala-Asp-Asp-Ala-Glu-Glu-Val-Glu-Gln-Lys-Gln-Leu-Gln |
| Endorphins | β-Endorphin | Endorphin, beta (Human) | Tyr-Gly-Gly-Phe-Met-Thr-Ser-Glu-Lys-Ser-Gln-Thr-Pro-Leu-Val-Thr-Leu-Phe-Lys-Asn-Ala-Ile-Ile-Lys-Asn-Ala-Tyr-Lys-Lys-Gly-Glu |
| | β-Endorphin (2-31) | Endorphin, beta (2-31) (Human) | Gly-Gly-Phe-Met-Thr-Ser-Glu-Lys-Ser-Gln-Thr-Pro-Leu-Val-Thr-Leu-Phe-Lys-Asn-Ala-Ile-Ile-Lys-Asn-Ala-Tyr-Lys-Lys-Gly-Glu- |
| | β-Endorphin (1-26) | Endorphin, beta (1-26) (Human) | Tyr-Gly-Gly-Phe-Met-Thr-Ser-Glu-Lys-Ser-Gln-Thr-Pro-Leu-Val-Thr-Leu-Phe-Lys-Asn-Ala-Ile-Ile-Lys-Asn-Ala |
| | β-Endorphin (1-27) | Endorphin, beta (1-27) (Human) | Tyr-Gly-Gly-Phe-Met-Thr-Ser-Glu-Lys-Ser-Gln-Thr-Pro-Leu-Val-Thr-Leu-Phe-Lys-Asn-Ala-Ile-Ile-Lys-Asn-Ala-Tyr |
| | β-Endorphin (rat) | Endorphin, beta (Rat) | Tyr-Gly-Gly-Phe-Met-Thr-Ser-Glu-Lys-Ser-Gln-Thr-Pro-Leu-Val-Thr-Leu-Phe-Lys-Asn-Ala-Ile-Ile-Lys-Asn-Val-His-Lys-Lys-Gly-Gln |
| | Ac - β-Endorphin (1-26) | acetyl-Endorphin, beta (1-26) (Human) | Ac-Tyr-Gly-Gly-Phe-Met-Thr-Ser-Glu-Lys-Ser-Gln-Thr-Pro-Leu-Val-Thr-Leu-Phe-Lys-Asn-Ala-Ile-Ile-Lys-Asn-Ala |
| | γ-Endorphin | Endorphin, gamma / Lipotropin, beta (61-77) (Human, Bovine, Ovine, Camel) | Tyr-Gly-Gly-Phe-Met-Thr-Ser-Glu-Lys-Ser-Gln-Thr-Pro-Leu-Val-Thr-Leu |
| Endomorphins | Endomorphin-1 | Endomorphin-1 | Tyr-Pro-Trp-Phe-NH2 |
| | Endomorphin-2 | Endomorphin-2 | Tyr-Pro-Phe-Phe-NH2 |
| Others | Thiorphan | Thiorphan / D,L-3-mercapto-2-benzylpropanoyl-Glycine | DL3-Mercapto-2-Benzylpropanoyl-Glycine |
| | Spinorphin | Spinorphin (Bovine) | Leu-Val-Val-Tyr-Pro-Trp-Thr |
| | Kyotorphin | Kyotorphin | Tyr-Arg |
| | Morphiceptin | Casomorphin (1-4), amide beta / Morphiceptin | Tyr-Pro-Phe-Pro-NH2 |
| | Morphiceptin PL017 | [N-MePhe, D-Pro4]-Morphiceptin / PL017 | Tyr-Pro-MePhe-D-Pro-NH2 |
| | Deltrophin | Dermenkephalin / Deltrophin (Phyllomedusa sauvageii) | Tyr-D-Met-Phe-His-Leu-Met-Asp-NH2 |
| | [D-Ala2]-Deltorphin I | [D-Ala2]-Deltorphin I | Tyr-D-Ala-Phe-Asp-Val-Val-Gly-NH2 |
| | [D-Ala2]-Deltorphin II | [D-Ala2]-Deltorphin II | Tyr-D-Ala-Phe-Glu-Val-Val-Gly-NH2 |
| | Hemorphin-4 | Hemorphin-4 (H-4) (Human, Bovine) | Tyr-Pro-Trp-Thr |
| | TIPP | TIPP / delta-Opioid Antagonist | Tyr-Tic-Phe-Phe |
| | [Y0-W2]-MIF-1 | [Tyr0, Trp2]-Melanocyte Inhibiting Factor 1 (MIF-1) | Tyr-Pro-Trp-Gly-NH2 |
| | [Y0]-MIF-1 | [Tyr0]-Melanocyte Inhibiting Factor 1 (MIF-1) | Tyr-Pro-Leu-Gly-NH2 |
| | β-Casomorphin 5 | Casomorphin (1-5), beta | Tyr-Pro-Phe-Pro-Gly |
| | CTOP amide | CTOP amide (Cys2, Tyr3, Orn5, Pen7) / Somatostatin Analog | D-Phe-Cys-Tyr-D-Trp-Orn-Thr-Pen-Thr-NH2 Disulfide bridge: Cys2-Pen7 |
| | CTAP amide | CTAP amide (Cys2, Tyr3, Arg5, Pen7) | D-Phe-Cys-Tyr-D-Trp-Arg-Thr-Pen-Thr-NH2 Disulfide bridge: Cys2-Pen7 |
| | TGF-1, alpha | Transforming Growth Factor 1 (TGF-1), alpha (Rat) | Tyr-D-Ala-Gly-N-Me-Phe-Gly-ol |

**Supplementary Table 2. Sequences of selected opioid peptides and their binding competition with CXCL12-AF647 on ACKR3 reported in Figure 1g and supplementary Figure 4a**

| Name | Sequence | Binding | |
|---|---|---|---|
| | | ACKR3 | |
| | | $IC_{50}$ (CI) nM | Min % |
| Dynorphin A | **YGGF**LRRIRPKLKWDNQ | 85.3 (64.2-118) | 6 |
| Dynorphin A 2-17 | **-GGF**LRRIRPKLKWDNQ | ~10,000 | 55* |
| Dynorphin A 2-13-NH2 | **-GGF**LRRIRPKLK-NH2 | > 10,000 | 75* |
| Dynorphin A 1-13 | **YGGF**LRRIRPKLK | 122 (96.3 - 162) | 9 |
| Dynorphin B | **YGGF**LRRQFKVVT | ~1000 | 33 |
| Leumorphin | **YGGF**LRRQFKVVTRSQEDPNAYSGELFDA | ~6000 | 41* |
| Big Dynorphin | **YGGF**LRRIRPKLKWDNQKR**YGGF**LRRQFKVVT | 32.8 (25.4 - 41.9) | 4 |
| Adrenorphin | **YGGF**MRRV-NH2 | 41.2 (28.2 - 60.1) | 7 |
| BAM22 | **YGGF**MRRVGRPEWWMDYQKRYG | 31.3 (27.6 - 35.5) | 3 |
| Met enkephalin | **YGGF**M | NB | 110* |
| Nociceptin | F**GGF**TGARKSARKLANQ | NB | 106* |
| Nociceptin 1-13-NH2 | F**GGF**TGARKSARK-NH2 | > 10,000 | 78* |
| [Phe1Ψ(CH2-NH)-gly2]nociceptin-(1-13)-NH2 | [Fψ(CH2-NH)G]GFTGARKSARK-NH2 | > 10,000 | 71* |
| Endomorphin-1 | YPWF-NH2 | > 10,000 | 81* |
| Endomorphin-2 | YPFF-NH2 | NB | 102* |
| β-endorphin | **YGGF**MTSEKSQTPLVTLFKNAIIKNAYKKGE | NB | 125* |
| LIH383 | F**GGF**MRRK-NH2 | 4.0 (3.1 - 5.1) | 1 |

$IC_{50}$ values are indicated in nanomolar (nM) with 95 % confidence interval (CI)

NB: No binding competition with 5 nM CXCL12-AF647

Min = minimal remaining AF647 signal measured at 3 µM non-labeled competitor and expressed as % of signal measured for 5 nM CXCL12-AF647 without competitor.

*measured at 9 µM

YGGF motif conserved in most of the opioid peptides is shown in bold. Positively charged residues are underlined

**Supplementary Table 3. Activity of positive controls used in Figure 2 and Supplementary Figure 2a**

| Receptor | Positive ctrl | β-arrestin-1 | β-arrestin-2 |
|---|---|---|---|
| | | Fold untreated ± SEM | Fold untreated ± SEM |
| **CCR1** | CCL3 | 2.01 ± 0.35 | 1.64 ± 0.14 |
| **CCR2B** | CCL2 | 9.49 ± 1.69 | 7.15 ± 1.05 |
| **CCR3** | CCL13 | 2.13 ± 0.19 | 1.88 ± 0.15 |
| **CCR4** | CCL22 | 8.20 ± 0.64 | 5.49 ± 0.14 |
| **CCR5** | CCL5 | 17.00 ± 0.77 | 9.07 ± 0.24 |
| **CCR6** | CCL20 | 13.26 ± 4.37 | 6.86 ± 0.52 |
| **CCR7** | CCL19 | 7.45 ± 1.27 | 5.94 ± 0.77 |
| **CCR8** | CCL1 | 2.91 ± 0.26 | 1.56 ± 0.28 |
| **CCR9** | CCL25 | 5.71 ± 1.38 | 5.69 ± 1.63 |
| **CCR10** | CCL27 | 4.97 ± 1.61 | 3.05 ± 0.52 |
| **CXCR1** | CXCL8 | 30.77 ± 5.68 | 14.54 ± 1.08 |
| **CXCR2** | CXCL8 | 2.46 ± 0.44 | 2.20 ± 0.24 |
| **CXCR3A** | CXCL11 | 8.16 ± 0.62 | 3.30 ± 0.17 |
| **CXCR3B** | CXCL11 | 6.05 ± 0.93 | 3.49 ± 0.36 |
| **CXCR4** | CXCL12 | 1.69 ± 0.04 | 1.37 ± 0.09 |
| **CXCR5** | CXCL13 | 4.85 ± 0.28 | 3.78 ± 0.10 |
| **CXCR6** | CXCL16 | 29.50 ± 5.45 | 16.52 ± 1.85 |
| **XCR1** | XCL2 | 4.75 ± 1.52 | 3.26 ± 1.07 |
| **CX3CR1** | CX3CL1 | 10.24 ± 1.72 | 8.66 ± 0.86 |
| **ACKR2** | CCL5 | 1.42 ± 0.09 | 1.33 ± 0.11 |
| **ACKR3** | CXCL12 | 3.03 ± 0.23 | 2.79 ± 0.36 |
| **ACKR4** | CCL19 | 6.02 ± 0.77 | 4.39 ± 0.54 |

Values correspond to fold increase over untreated and represent the mean of three independent experiments ± standard error of the mean.

**Supplementary Table 4. Sequences of adrenophin-derived peptides and activity in β-arrestin-1 recruitment towards human opioid receptors reported in Figure 3.**

| Sequence | Position | Receptor | | | |
|---|---|---|---|---|---|
| | | MOR | DOR | KOR | ACKR3 |
| | | $EC_{50}$ (CI) nM | $EC_{50}$ (CI) nM | $EC_{50}$ (CI) nM | $EC_{50}$ (CI) nM |
| YGGFMRRV | Ref. | 101 (83.0 - 125) | 323 (258 - 409) | 65.6 (56.5 – 75.9) | 109 (70.7 – 182) |
| **K**GGFMRRV | 1 | NA | NA | NA | NA |
| **A**GGFMRRV | 1 | NA | NA | NA | >10,000 |
| **F**GGFMRRV | 1 | NA | NA | NA | 9.33 (5.12 – 16.0) |
| **N**GGFMRRV | 1 | NA | NA | NA | NA |
| **L**GGFMRRV | 1 | NA | NA | NA | 261 (nc – 573) |
| Y**A**GFMRRV | 2 | 1094 (776 – 4207) | 2317 (nc – 75020) | NA | 611 (397 – 1052) |
| Y**S**GFMRRV | 2 | NA | NA | NA | ~10,000 |
| Y**R**GFMRRV | 2 | NA | NA | NA | ~5,000 |
| YG**A**FMRRV | 3 | >10,000 | 1581 (nc – 4986) | 315 (253 – 392) | 263 (163 – 425) |
| YG**S**FMRRV | 3 | >10,000 | >10,000 | >10,000 | 668 (465 – 1049) |
| YGG**Y**MRRV | 4 | NA | NA | NA | ~5,000 |
| YGG**W**MRRV | 4 | 522 (361 – 725) | 1018 (844 – 1436) | >10,000 | 70.3 (38.3 – 136) |
| YGG**L**MRRV | 4 | NA | NA | NA | ~10,000 |
| YGG**A**MRRV | 4 | NA | NA | NA | ~10,000 |
| YGGF**L**RRV | 5 | 880 (624 – 1572) | 513 (403 – 639) | 31.9 (28.4 – 25.8) | 684 (368 – 1682) |
| YGGF**A**RRV | 5 | >10,000 | >10,000 | 656 (555 -775) | ~5,000 |
| YGGF**K**RRV | 5 | NA | NA | NA | ~10,000 |
| YGGF**Q**RRV | 5 | ~5000 | >10,000 | 283 (221 – 366) | ~5,000 |
| YGGF**F**RRV | 5 | 363 (225 – nc) | 1313 ( nc – 5420) | 194 (81.3 – 900) | 1535 ( nc – 4196) |
| YGGF**N**RRV | 5 | NA | NA | 508 (441 – 586) | ~10,000 |
| YGGFM**A**RV | 6 | 35.4 (27.7 – 45.2) | 147 (98.5 – 238) | 2238 (nc – 12290) | ~5,000 |
| YGGFM**K**RV | 6 | 63.8 (50.6 – 79.3) | 360 (248 – 533) | 416 (355 – 485) | ~5,000 |
| YGGFMR**A**V | 7 | 682 (227 – nc) | 308 (214 – 461) | 1250 (1001 – 2131) | NA |
| YGGFMR**K**V | 7 | 786 (567 – 1250) | 378 (260 – 565) | 230 (212 – 251) | ~10,000 |
| YGGFMRR**L** | 8 | 294 (235 – 366) | 254 (184 – 354) | 71.2 (59.6 – 84.5) | 153 (104 – 242) |
| YGGFMRR**I** | 8 | 188 (nc – 260) | 320 (215 – 470) | 76.9 (55.9 – 105) | 115 (81.1 – 177) |
| YGGFMRR**A** | 8 | 265 (nc – 448) | 260 ( nc – 427) | 132 (59.8 – 343) | 201 (109 – 478) |
| YGGFMRR**F** | 8 | 198 (177 – 220) | 212 (171 – 261) | 80.9 (66.9 – 96.5) | 73.5 (45.2 – 119) |
| YGGFMRR**K** | 8 | 252 (187 – 342) | 527 (383 – 713) | 19.2 (14.9 – 24.8) | 37.0 (19.1 – 75.0) |
| YGGFMRR**Q** | 8 | 183 (143 – 240) | 259 (180 – 381) | 63.1 (53.0 – 74.6) | 110 (43.6 – 499) |
| **-** GGFMRRV | 0 | NA | NA | NA | >10,000 |
| **A**YGGFMRRV | 0 | 308 (238 – 396) | 637 (240 – 19890) | 205 (157 – 270) | 330 (nc – 1394) |
| YGGFMRRV**A** | 9 | 174 ( nc – 270) | 272 (186 – 403) | 57.5 (49.6 – 66.2 | 72.3 (50.7 – 101) |
| YGGFMRRV**R** | 9 | 58.7 (46.1 – 73.5) | 193 (136 – 283) | 21.9 (20.3 – 23.7) | 12.6 (7.47 – 21.2) |
| yggfmrrv | Daa | NA | NA | NA | >10.000 |
| (YGGFMRRVC)2 | dimer | 271 (188 – 389) | 253 (154 – 337) | 79.4 (44.7 – 138) | 52.9 (17.9 – 173) |

$EC_{50}$ values are indicated in nanomolar (nM) with 95 % confidence interval (CI)

NA: No activity or activity below 10 % of positive control in the concentration range tested

nc; not calculable

Note that all peptides showed no activity towards NOP

Peptides presented in Figure 3 b-e are marked in red

**Supplementary Table 5. Potencies of peptide variants combining mutation Y1F with other modifications in β-arrestin-1 recruitment to ACKR3.**

| 2nd generation peptides | ACKR3 | |
|---|---|---|
| **Sequence** | **EC50 (nM)** | **pEC50 ± SEM** |
| FGGFMRR**K** (LIH383) | 0.61 | 9.21 ± 0.17 |
| FGGFMRR**KR** | 1.12 | 8.96 ± 0.20 |
| FGGFMRRV**R** | 3.53 | 8.45 ± 0.10 |
| FGG**W**MRR**K** | 4.39 | 8.36 ± 0.12 |
| FGG**W**MRRV**R** | 6.16 | 8.21 ± 0.14 |
| FGG**W**MRR**KR** | 7.34 | 8.14 ± 0.13 |
| FGG**W**MRRV | 10.52 | 7.98 ± 0.62 |
| FGGFMRR**F** | 10.96 | 7.96 ± 0.29 |
| FGGFMRR**FR** | 16.34 | 7.79 ± 0.24 |
| FGG**W**MRR**FR** | 19.07 | 7.72 ± 0.22 |
| FGG**W**MRR**F** | 19.10 | 7.72 ± 0.20 |
| FGGFMRRV (Y1F adrenorphin) | 9.33 | 8.03 ± 0.11 |
| YGGFMRRV (adrenorphin) | 108.7 | 6.96 ± 0.09 |
| MRRKFGGF (LIH383 ctrl) | inactive | |

Modified amino acids with respect to peptide Y1F-adrenorphin (FGGFMRRV) are underlined and in bold

**Supplementary Table 6. Activity of LIH383, adrenorphin and chemokines in β-arrestin-1 recruitment to human and mouse ACKR3 and classical human opioid receptors reported in Figure 4 b-f**

| Name | β-arrestin-1 | | | | | | | | | | | |
|---|---|---|---|---|---|---|---|---|---|---|---|---|
| | hACKR3 | | MOR | | DOR | | KOR | | NOP | | mACKR3 | |
| | $EC_{50}$ (CI) nM | Max % | $EC_{50}$ (CI) nM | Max % | $EC_{50}$ (CI) nM | Max % | $EC_{50}$ (CI) nM | Max % | $EC_{50}$ (CI) nM | Max % | $EC_{50}$ (CI) nM | Max % |
| LIH383 | 0.61 (0.19 – 1.17) | 99 | NA | 1 | NA | 1 | NA | 9 | NA | 1 | 2.31 (0.008 – 7.56) | 95 |
| LIH383 Ctrl | NA | 4 | - | - | - | - | - | - | - | - | ND | 17 |
| CXCL12 | 1.21 (0.83 – 1.74) | 100 | - | - | - | - | - | - | - | - | - | - |
| CXCL11 | 2.20 (0.78 – 4.81) | 75 | - | - | - | - | - | - | - | - | - | - |
| Adrenorphin | 56.5 (36.6 – 95.0) | 96 | 41.6 (30.7 – 57.7) | 96 | 157 (104 – 310) | 96 | 43.5 (33.7 – 57.9) | 98 | NA | 3 | - | - |
| mCXCL12 | - | - | - | - | - | - | - | - | - | - | 3.32 (1.17 – 33.6) | 100 |
| LIH383-Cy5 | 6.65 (5.52 – 8.29) | 100 | - | - | - | - | - | - | - | - | - | - |

$EC_{50}$ values are indicated in nanomolar (nM) with 95 % confidence interval (CI)

ND: Not determinable since saturation was not reached

NA: No activity or activity below 10 % of positive control in the concentration range tested

Max = maximum signal measured at 3 μM expressed as % of the reference peptide

- = not analyzed

**Supplementary Table 7. Activity of selected opioid peptides in mini Gi recruitment to ACKR3, MOR, KOR, DOR and NOP reported in Figure 5e.**

| Name | Sequence | mini Gi | | | | | | | | | |
|---|---|---|---|---|---|---|---|---|---|---|---|
| | | ACKR3 | | MOR | | DOR | | KOR | | NOP | |
| | | EC50 (CI) nM | Max % | EC50 (CI) nM | Max % | EC50 (CI) nM | Max % | EC50 (CI) nM | Max % | EC50 (CI) nM | Max % |
| CXCL12 | | NA | NA | NA | NA | NA | NA | NA | NA | NA | NA |
| BAM22 | **YGGF**MRRVGRPEWWMDYQKRYG | NA | NA | 17.8 (12.2 – 26.5) | 100 | 1324 (547 - nc) | 63 | - | - | - | - |
| Adrenorphin | **YGGF**MRRV-NH2 | NA | NA | 38.0 (14.23 – 43840) | 110 | 315 (92.6 - nc) | 89 | - | - | - | - |
| Met enkephalin | **YGGF**M | NA | NA | 170 (97.7 – 453) | 89 | 39.4 (11.7 - nc) | 100 | - | - | - | - |
| Dynorphin A | **YGGF**LRRIRPKLKWDNQ | NA | NA | - | - | - | - | 11.8 (6.49 – 22.0) | 100 | - | - |
| Dynorphin B | **YGGF**LRRQFKVVT | NA | NA | - | - | - | - | 6.06 (1.99 – 14.8) | 95 | - | - |
| Big Dynorphin | **YGGF**LRRIRPKLKWDNQKR**YGGF**LRRQFKVVT | NA | NA | - | - | - | - | 16.1 (8.98 – 28.7) | 94 | - | - |
| Nociceptin | F**GGF**TGARKSARKLANQ | NA | NA | - | - | - | - | - | - | 5.97 (2.45 – 12.3) | 100 |
| Nociceptin 1-13-NH2 | F**GGF**TGARKSARK-NH2 | NA | NA | - | - | - | - | - | - | 1.06 (0.008 – 2.97) | 108 |
| [Phe1Ψ(CH2-NH)-gly2]nociceptin-(1-13)-NH2 | [Fψ(CH2-NH)G]GFTGARKSARK-NH2 | NA | NA | - | - | - | - | - | - | 32.2 (21.6 – 53.8) | 33 |
| β-endorphin | **YGGF**MTSEKSQTPLVTLFKNAIIKNAYKKGE | NA | NA | 1021 (342 - nc) | 64 | 931 (208 - nc) | 70 | - | - | - | - |
| Endomorphin-1 | YPWF-NH2 | NA | NA | 162 (37.9 - nc) | 66 | - | - | - | - | - | - |
| Endomorphin-2 | YPFF-NH2 | NA | NA | 175 (53.4 - nc) | 63 | - | - | - | - | - | - |

$EC_{50}$ values are indicated in nanomolar (nM) with 95 % confidence interval (CI)

NA: No activity or activity below 10 % of positive control in the concentration range tested

Max = maximum signal measured at 3 µM expressed as % of the reference peptide

nc: not calculable

- = not analyzed

YGGF motif conserved in most of the opioid peptides is shown in bold. Positively charged residues are underlined

**Supplementary Table 8. Sequences and annealing temperatures of qPCR primers used for receptor mRNA quantification in human brain samples and smNPC represented in Figure 7b and 7c.**

| Gene | $T_a$ | Primer Sequence | Reference |
|---|---|---|---|
| *ACKR3* | 57°C | F: 5'-TCGGCAGCATTTTCTTCCTC-3'<br>R: 5'-GCAGTAGGTCTCATTGTTGGAC-3' | *Ikeda et al, 2013*[1] |
| *OPRD* | 60°C | F: 5'-TCGGGCTCCAAGGAGAAG-3'<br>R: 5'-TCGTCGAGGAAAGCGTAGA-3' | *Ikeda et al, 2013*[1] |
| *OPRK* | 61°C | F: 5'-CTGCATCTGGCTGCTGTCGT-3'<br>R: 5'-GACGCAGGATCATCAGGGTGTAG-3' | *Ikeda et al, 2013*[1] |
| *OPRM* | 58°C | F: 5'-CCGTGTGCTATGGACTGA-3'<br>R: 5'-GAAATGCATAAAGGACTGGGTT-3' | *Ikeda et al, 2013*[1] |
| *OPRL* | 62°C | F: 5'-CTCGGCTGGTGCTGGTGGTA-3'<br>R: 5'-CGTGCAGAAGCGCAGAATGG-3' | *Li et al, 2013*[2] |
| *PPIA* | 60°C | F: 5'-TCCTGGCATCTTGTCCATG-3'<br>R: 5'-CCATCCAACCACTCAGTCTTG-3' | *Schote et al, 2007*[3] |
| *GAPDH* | 60°C | F: 5'-GAAGGTGAAGGTCGGAGTC-3'<br>R: 5'-GAAGATGGTGATGGGATTTC-3' | *Schote et al, 2007*[3] |

All primer pairs have been validated and used in previous studies (see references[1, 2, 3])

**Supplementary Table 9. Sequences of selected opioid peptides and their activity in β-arrestin-1 or β-arrestin-2 recruitment to ACKR3 in different cell lines reported in supplementary Figure 1 b-d.**

| Name | Sequence | β-arrestin-2 | | β-arrestin-1 | | | |
|---|---|---|---|---|---|---|---|
| | | ACKR3-U87 | | ACKR3-HEK | | ACKR3-CHO | |
| | | $EC_{50}$ (CI) nM | Max % | $EC_{50}$ (CI) nM | Max % | $EC_{50}$ (CI) nM | Max % |
| Dynorphin A | **YGGF**LRRIRPKLKWDNQ | 97.6 (70.5 – 138) | 111 | 110 (97.2 – 125) | 89 | 70.5 (54.5 – 93.9) | 107 |
| Dynorphin B | **YGGF**LRRQFKVVT | 908 (373 - nc) | 88 | 673 (572 – 831) | 74 | ~1000 | 96 |
| Leumorphin | **YGGF**LRRQFKVVTRSQEDPNAYSGELFDA | 902 (342 - nc) | 92* | 2708 (1302 – 92760) | 65* | 1723 (700 - nc) | 88* |
| Big Dynorphin | **YGGF**LRRIRPKLKWDNQKR**YGGF**LRRQFKVVT | 76.6 (54.9 – 109.2) | 132 | 60.7 (52.9 – 69.8) | 87 | 47.5 (36.1 – 61.5) | 100 |
| Adrenorphin | **YGGF**MRRV-NH2 | 81.5 (40.1 – 342) | 93 | 72.6 (51.1 – 114) | 69 | 22.5 (15.0 – 34.8) | 81 |
| BAM22 | **YGGF**MRRVGRPEWWMDYQKRYG | 22.9 (15.4 – 35.6) | 106 | 23.2 (17.8 – 30.6) | 75 | 12.5 (9.10 – 19.6) | 93 |
| Met enkephalin | **YGGF**M | ND | 13* | NA | 0* | NA | 1* |
| Nociceptin | F**GGF**TGARKSARKLANQ | >10,000 | 43* | ND | 24* | ~10,000 | 55* |
| Nociceptin 1-13-NH2 | F**GGF**TGARKSARK-NH2 | ~10,000 | 68* | ~10,000 | 47* | ~10,000 | 65* |
| [Phe1Ψ(CH2-NH)-gly2]nociceptin-(1-13)-NH2 | [Fψ(CH2-NH)G]GFTGARKSARK-NH2 | 4344 (1429 - nc) | 92* | 3761 (2339 – 9706) | 82* | ~5000 | 97* |
| Endomorphin-1 | YPWF-NH2 | ND | 34* | ND | 13* | ND | 17* |
| Endomorphin-2 | YPFF-NH2 | ND | 24* | ND | 7* | ND | 3* |
| β-endorphin | **YGGF**MTSEKSQTPLVTLFKNAIIKNAYKKGE | ND | 28* | ND | 5* | ND | 7* |

$EC_{50}$ values are indicated in nanomolar (nM) with 95 % confidence interval (CI)

ND: Not determinable since saturation was not reached

NA: No activity or activity below 10 % of positive control in the concentration range tested

nc: not calculable

Max = maximum signal measured at 3 µM expressed as % of signal measured for CXCL12

*measured at 9 µM

YGGF motif conserved in most of the opioid peptides is shown in bold. Positively charged residues are underlined

**Supplementary Table 10. Off-target activity of selected opioid peptides in β-arrestin-1 and β-arrestin-2 recruitment towards human chemokine receptors reported in supplementary Figure 2b**

| Receptor | β-arrestin-1 | | | | β-arrestin-2 | | | |
|---|---|---|---|---|---|---|---|---|
| | Positive Ctrl | | Big Dynorphin | | Positive Ctrl | | Big Dynorphin | |
| | chemokine – $EC_{50}$ (CI) nM | Max | $EC_{50}$ (CI) nM | Max | chemokine – $EC_{50}$ (CI) nM | Max | $EC_{50}$ (CI) nM | Max |
| **CXCR3A** | CXCL11 – 5.14 (2.03 – 72.7) | 6.93 | ND | 1.83 | CXCL11 – 8.65 (4.26 – 57.1) | 3.65 | ND | 1.43 |
| **CXCR3B** | CXCL11 – 25.6 (16.1 – 61.8) | 6.71 | ND | 1.57 | CXCL11 – 40.2 (14.1 – nc) | 3.40 | ND | 1.42 |
| **CX3CR1** | CX3CL1 – 0.02 (0.000005 – 0.06) | 14.00 | ND | 2.20 | CX3CL1 – 0.04 (0.0002 – 0.12) | 7.09 | ND | 1.68 |
| **CCR3** | CCL13 – 30.1 (15.5 – 195) | 3.41 | ND | 1.47 | CCL13 – 28.7 (11.2 – 13220) | 3.05 | ND | 1.23 |

$EC_{50}$ values are indicated in nanomolar (nM) with 95 % confidence interval (CI)

ND: Not determinable since saturation was not reached

Max = signal measured at 300 nM for chemokines or 3 µM for big dynorphin expressed as fold untreated

nc: not calculable

## Supplementary References


1. Ikeda Y, Kumagai H, Skach A, Sato M, Yanagisawa M. Modulation of circadian glucocorticoid oscillation via adrenal opioid-CXCR7 signaling alters emotional behavior. *Cell* **155**, 1323-1336 (2013).

2. Li L, Dong L, Wang S. Expression of the nociceptin/orphanin FQ receptor in the intestinal mucosa of IBS patients. *Exp Ther Med* **6**, 679-683 (2013).

3. Schote AB, Turner JD, Schiltz J, Muller CP. Nuclear receptors in human immune cells: expression and correlations. *Mol Immunol* **44**, 1436-1445 (2007).
