# Supplementary material for: The atypical chemokine receptor ACKR3/CXCR7 is a broad-spectrum scavenger for opioid peptides
Source: Nat Commun. 2020 Jun 19;11:3033. doi: 10.1038/s41467-020-16664-0 (PMC7305236; doi:10.1038/s41467-020-16664-0)
Supplement: Supplementary file 3 — Reporting Summary [file 41467_2020_16664_MOESM3_ESM.pdf]

nature research

Corresponding author(s): Andy Chevigné

Last updated by author(s): May 6, 2020

Reporting Summary

Nature Research wishes to improve the reproducibility of the work that we publish. This form provides structure for consistency and transparency in reporting. For further information on Nature Research policies, see Authors & Referees and the Editorial Policy Checklist .

Statistics

For all statistical analyses, confirm that the following items are present in the figure legend, table legend, main text, or Methods section.

n/a	Confirmed	
☐	☒	The exact sample size (*n*) for each experimental group/condition, given as a discrete number and unit of measurement
☐	☒	A statement on whether measurements were taken from distinct samples or whether the same sample was measured repeatedly
☐	☒	The statistical test(s) used AND whether they are one- or two-sided<br>*Only common tests should be described solely by name; describe more complex techniques in the Methods section.*
☒	☐	A description of all covariates tested
☐	☒	A description of any assumptions or corrections, such as tests of normality and adjustment for multiple comparisons
☐	☒	A full description of the statistical parameters including central tendency (e.g. means) or other basic estimates (e.g. regression coefficient) AND variation (e.g. standard deviation) or associated estimates of uncertainty (e.g. confidence intervals)
☐	☒	For null hypothesis testing, the test statistic (e.g. *F*, *t*, *r*) with confidence intervals, effect sizes, degrees of freedom and *P* value noted<br>*Give P values as exact values whenever suitable.*
☒	☐	For Bayesian analysis, information on the choice of priors and Markov chain Monte Carlo settings
☒	☐	For hierarchical and complex designs, identification of the appropriate level for tests and full reporting of outcomes
☒	☐	Estimates of effect sizes (e.g. Cohen's *d*, Pearson's *r*), indicating how they were calculated

Our web collection on statistics for biologists contains articles on many of the points above.

Software and code

Policy information about availability of computer code

Data collection	Flow cytometry: BD FACS Diva 8.01 (BD Biosciences) or NovoExpress 1.4.1 (ACEA Biosciences)<br>Imaging flow cytometry: INSPIRE Mark II (EMD Millipore);<br>Nanoluciferase-based and HTRF assays: MicroWin2010 5.19 (Mikrotek Laborsysteme);<br>qPCR: Bio-Rad CFX Manager 3.1;<br>Neuron depolarization experiments: Spike2 (CED);<br>Confocal microscopy: Zen Black 2.3 SP1 (Zeiss)<br>GPCR data base www.gpcrdb.org was used to generate receptor snake diagram, conserved residue comparison and the phylogenetic tree presented in Supplementary Figure 3.<br>CNS gene expression data were extracted from Allen Institute, BrainSpan: Atlas of the Developing Human Brain www.brainspan.org
Data analysis	Statistical analysis and curve fitting: GraphPad Prism 8.0.1;<br>Flow cytometry: Flowjo 10.6.1<br>Imaging flow cytometry: IDEAS 6.2;<br>qPCR: Bio-Rad CFX Manager 3.1;<br>Neuron depolarization experiments: Spike2 (CED);<br>Confocal microscopy: Zen Black 2.3 SP1 (Zeiss)<br>Sequence alignment and phylogenetic tree formating: CLC main workbench 7.9.1;<br>Numerical data processing: Microsoft Excel 2016;

For manuscripts utilizing custom algorithms or software that are central to the research but not yet described in published literature, software must be made available to editors/reviewers. We strongly encourage code deposition in a community repository (e.g. GitHub). See the Nature Research guidelines for submitting code & software for further information.

Data

Policy information about availability of data

All manuscripts must include a data availability statement. This statement should provide the following information, where applicable:

- Accession codes, unique identifiers, or web links for publicly available datasets
- A list of figures that have associated raw data
- A description of any restrictions on data availability

All data supporting the findings of this study are available in the Article and its Supplementary Information or from the corresponding author upon reasonable request.
Raw data is provided as separate excel file for all numerical data of figures 1-7 and supplementary figures 1-6
Central nervous system gene expression data were extracted from Allen Institute, BrainSpan: Atlas of the Developing Human Brain http://www.brainspan.org/static/download.html
GPCR data base www.gpcrdb.org was used to generate receptor snake diagram, conserved residue comparison and the phylogenetic tree presented in Supplementary Figure 3.

Field-specific reporting

Please select the one below that is the best fit for your research. If you are not sure, read the appropriate sections before making your selection.

☒ Life sciences ☐ Behavioural & social sciences ☐ Ecological, evolutionary & environmental sciences

For a reference copy of the document with all sections, see nature.com/documents/nr-reporting-summary-flat.pdf

Life sciences study design

All studies must disclose on these points even when the disclosure is negative.

Sample size	Sample sizes and number of replicates were chosen so as to allow to demonstrate reproducibility of the measurements and generate statistically significant results. In preliminary experiments comparing the effect of dynorphin alone and dynorphin + 3 µM LIH, we observed large differences in EC50 with values around 350 and 120 nM, respectively. Assuming a variability yielding a SD of approximately 1/3 of the mean (typically obtained for concentration-response curves done in rat brain slices), the predicted effect size was ~(350-120)/117~ 2. A G*Power analysis showed that, with such a large effect size, a sample of 6 yields a statistical power > 95%.
Data exclusions	In the ex vivo rat brain sample analysis, one value, which was > 2SD away from the mean for its experimental group, was excluded. This aberrant value presumably resulted from later-revealed inadequate storage conditions of dynorphin A used for this particular experiment. The measurement was repeated with a new batch of dynorphin A and the value was consistent with other results obtained for the experimental group.
Replication	For all in vitro studies, each experiment was repeated at least three times. For ex vivo studies, six different rat brains were used per treatment in independent experiments. All experiments could be reliably reproduced with little variability between the experiments (see S.E.M for the respective experiments), with the exception of one brain sample (see Data exclusion section above).
Randomization	For ex vivo studies, all rats were male at roughly the same age and weight. They were randomly assigned to experimental treatment groups.
Blinding	The investigators were not blinded during data collection and analysis. The majority of data collected are quantitative and not subject to investigator bias.

Reporting for specific materials, systems and methods

We require information from authors about some types of materials, experimental systems and methods used in many studies. Here, indicate whether each material, system or method listed is relevant to your study. If you are not sure if a list item applies to your research, read the appropriate section before selecting a response.

Materials & experimental systems

n/a	Involved in the study	
☐	☒	Antibodies
☐	☒	Eukaryotic cell lines
☒	☐	Palaeontology
☐	☒	Animals and other organisms
☐	☒	Human research participants
☒	☐	Clinical data

Methods

n/a	Involved in the study	
☒	☐	ChIP-seq
☐	☒	Flow cytometry
☒	☐	MRI-based neuroimaging

Antibodies

Antibodies used	ACKR3-specific antibodies:<br>clone 11G8 (R&D Systems, Cat # MAB42273, Lot YQU0217071)<br><br>KOR-specific antibody:<br>clone 387301 (R&D Systems, Cat # MAB3895, Lot YNO0217111)<br><br>IgG1, k isotype control:<br>clone MG1-45 (BioLegend Cat # 401402)<br><br>All non-conjugated antibodies were combined with a secondary R-Phycoerythrin AffiniPure F(ab')$_2$ Fragment Goat Anti-Mouse IgG (H+L) (Jackson ImmunoResearch, Cat # 115-116-146, Lot: 124434))
Validation	The antibodies recognized cells transfected with ACKR3- or KOR-encoding vectors but not untransfected cells. A corresponding isotype control was used as additional control and for cells endogenously expressing ACKR3.<br><br>These antibodies are commercially available and routinely used by many researchers.<br>All antibodies are validated by the manufacturer:<br><br>clone 11G8: Detects human CXCR7/RDC-1 in flow cytometry of five distinct human CXCR7 transfectants, but not their respective parental lines. (https://www.rndsystems.com/products/human-cxcr7-rdc-1-antibody-11g8_mab42273)<br><br>clone 387301: Detects human KOR. Stains human KOR transfectants but not irrelevant transfectants. (https://www.rndsystems.com/products/human-kor-antibody-387301_mab3895)<br><br>clone MG1-45: The isotype of MG1-45 immunoglobulin is a mouse IgG1, κ. It was chosen as isotype control after screening on a variety of resting, activated, live, and fixed mouse, rat and human tissues. (https://www.biolegend.com/en-us/products/purified-mouse-igg1--kappa-isotype-ctrl-2621)<br><br>R-Phycoerythrin AffiniPure F(ab')$_2$ Fragment Goat Anti-Mouse IgG: Based on immunoelectrophoresis and/or ELISA, the antibody reacts with whole molecule mouse IgG. It also reacts with the light chains of other mouse immunoglobulins. No antibody was detected against non-immunoglobulin serum proteins. (www.jacksonimmuno.com/catalog/products/115-116-146)

Eukaryotic cell lines

Policy information about cell lines

Cell line source(s)	U87 cells derived from human glioblastoma were obtained through the NIH AIDS Reagent Program.<br>HEK293T and CHO-K1 cells were obtained from ATCC.<br>Small molecule neural precursor cells (smNPCs, cell line C1-1) were derived from a healthy male at age 67, first characterized and used in the following publication: Schondorf, D. C. et al. Nat Commun. 2014;5:4028 (2014)
Authentication	The cell lines used were not authenticated in our laboratories.
Mycoplasma contamination	All cell lines were regularly tested for mycoplasma contamination using the Venor GeM OneStep kit (Minerva BioLabs). None of the cell lines were ever contaminated.
Commonly misidentified lines<br>(See ICLAC register)	No commonly misidentified lines were used in this study.

Animals and other organisms

Policy information about studies involving animals; ARRIVE guidelines recommended for reporting animal research

Laboratory animals	6-8-week old Wistar male rats were used in the study.
Wild animals	The study did not involve wild animals.
Field-collected samples	The study did not involve samples collected from the field.
Ethics oversight	All procedures were carried out in accordance with the guidelines of the European Communities Council Directive of 24 November 1986 (86/609/EE) and were accepted by the Ethics Committee for Animal Use of the University of Liège (protocol 2061).

Note that full information on the approval of the study protocol must also be provided in the manuscript.

Human research participants

Policy information about studies involving human research participants

Population characteristics	Post-mortem brain tissues were obtained from five donors (four males and one female) from Thailand, aged between 20 and 59 years, with no known underlying disease, who had been hospitalized after accidents excluding head or brain injuries, to which they eventually succumbed.<br><br>Small-molecule neural precursor cells (smNPCs, cell line C1-1) were derived from a healthy male at age 67, whose informed consent was obtained, first characterized and used in the following publication: Schondorf, D. C. et al. Nat Commun. 2014;5:4028 (2014)
Recruitment	Post mortem brain tissues were obtained from five donors from a small, well defined, geographic location in and around Chang Mai in Northern Thailand, with no known underlying disease who had been hospitalised after accidents excluding head or brain injuries, to which they eventually succumbed at the Intensive Care Unit of the Chiang Mai University Medical Hospital. There was little inclusion bias as these post-mortem samples were collected within 2-10 hours after death to maintain RNA integrity. Brain autopsies were performed for medical or forensic analysis after informed consent of the closest relatives. Informed consent was also given for the use of anonymized brain tissues and clinical and pathological information for research purposes are available in Cao-lei et al. J Psychiatr Res 47, 1597-1607, (2013)
Ethics oversight	The study was approved by the Ethical Committee of the Faculty of Medicine, Chiang Mai University, Thailand (protocol 2012-038).

Note that full information on the approval of the study protocol must also be provided in the manuscript.

Flow Cytometry

Plots

Confirm that:

- [x] The axis labels state the marker and fluorochrome used (e.g. CD4-FITC).
- [x] The axis scales are clearly visible. Include numbers along axes only for bottom left plot of group (a 'group' is an analysis of identical markers).
- [x] All plots are contour plots with outliers or pseudocolor plots.
- [x] A numerical value for number of cells or percentage (with statistics) is provided.

Methodology

Sample preparation	For U87 cell lines: cells were detached with PBS/EDTA 0.48 mM, harvested in Opti-MEM and distributed into 96-well plates.<br>For ligand uptake assays: after the indicated treatment, cells were washed twice with FACS buffer (PBS 1X 1% BSA, 0.1% NaN2). Subsequently, cells were incubated 30 minutes at 4°C with Zombie viability dye (BioLegend). Cells were then washed once and resuspended in FACS buffer for analysis.<br>For receptor staining: After indicated treatment, ligands were removed by washing with 150 mM NaCl, 50 mM glycine, pH 3 and twice with FACS buffer. Cells were then incubated for 45 minutes with primary antibody at 4°C in FACS buffer, then after a washing step, incubated for 30 minutes with PE-conjugated secondary antibody and Zombie viability dye (BioLegend). Cells were then washed once and resuspended in FACS buffer for analysis.<br>For ACKR3 staining on smNPC cells, cells were detached with accutase and distributed into 96-well plates. Staining steps with antibodies were as described above.<br>For intracellular staining, cells were treated with Fixation/Permeabilization Solution Kit (BD Biosciences, Catalog No. 554714). Other preparation steps were as described above, except that no viability dye was used and that antibody incubation was done in Perm/Wash buffer.
Instrument	BD FACS LSR Fortessa cytometer (BD Biosciences) or Novocyte Quanteon (Acea BioSciences)<br>The Amnis brand ImageStreamX Mark II (EMD Millipore) was used for imaging flow cytometry
Software	For flow cytometry BD FACSDiva 8.01 and NovoExpress 1.4.1 were used.<br>For imaging flow cytometry INSPIRE Mark II (EMD Millipore) and IDEAS 6.2 (EMD Millipore) softwares were used for data acquisition and analysis, respectively.
Cell population abundance	Flow and imaging cytometry were performed on U87 cell line or smNPCs stably expressing the receptor of interest with uniform expression in the entire cell population. For figure 6b, cells were transfected with equal amounts of GPCR-encoding pcDNA plasmids and the entire cell population was used for analysis.

Gating strategy

Flow and imaging cytometry were used to monitor GPCR expression and the binding/internalization of fluorescently labeled ligands to cells overexpressing the receptor of interest. For flow cytometry, viable cells were determined based on size and granularity (SSC-H/FSC-H) and viablity dye. Doublets and coincident events were excluded (SSC-H/SSC-A). For imaging flow cytometry, single (Area/Aspect Ratio), in-focus (Gradient RMS) and living (viability dye) cells were selected. Fluorescent ligand binding to viable single cells or receptor expression were quantified based on mean fluorescence intensity. 10,000 gated cells were acquired.

☒ Tick this box to confirm that a figure exemplifying the gating strategy is provided in the Supplementary Information.
